# DISTAL NEUROTIZATION OF THE ANTERIOR INTEROSSEOUS NERVE TO RECOVER HAND GRASPING

**DOI:** 10.1590/1413-785220233101e257852

**Published:** 2023-02-20

**Authors:** ÁLVARO BAIK CHO, CARLOS HENRIQUE VIEIRA FERREIRA, RENAN MARTINS FONTANA, GARY ALAN ÂNGULO MONTANO, LEANDRO YOSHINOBU KIYOHARA, LUIZ SORRENTI

**Affiliations:** 1 Faculdade de Medicina do ABC, Hand and Microsurgery Group, Santo André, SP, Brazil.; 2 Universidade de São Paulo, School of Medicine, Department of Orthopedics and Traumatology, Hand and Microsurgery Group, São Paulo, SP, Brazil.

**Keywords:** Nerve Transfer, Microsurgery, Nerve Lesion, Transferência de Nervo, Microcirurgia, Lesão do Nervo

## Abstract

Lower trunk lesions are uncommon, representing about 3 to 5% of brachial plexus lesions in adults. One of the functions lost by patients who suffer this type of injury is the flexion of the fingers, with important harming of palmar grip. This series of cases proposes the transfer of a branch of the radial nerve to the anterior interosseous nerve (AIN), presenting a new alternative for the treatment of these lesions with highly satisfactory results.

**Objective:**

To demonstrate our strategy, technique, and results in the reinnervation of the AIN in lesions isolated from the lower trunk of the brachial plexus in four cases of high lesion of the median nerve.

**Method:**

Prospective cohort study in which four patients underwent neurotizations. The treatment was directed to the recovery of the fingers’ flexors of the hand and the grip.

**Results:**

All patients presented reinnervation of the flexor pollicis longus (FPL) and deep flexors of the 2nd, 3rd, and 4th fingers. The deep flexor of the 5th finger also showed reinnervation but with reduced strength (M3/4) comparing to the others (M4+).

**Conclusion:**

Despite the limited number of cases in this and other studies, the results are uniformly good, allowing to consider this treatment predictable. ***Level of Evidence IV, Case Series.***

## RESUMO

As lesões do tronco inferior são incomuns, representando cerca de 3 a 5% das lesões do plexo braquial em adultos. Uma das funções perdidas pelos pacientes que sofrem esse tipo de lesão é a flexão dos dedos, com comprometimento importante da preensão palmar. Esta série de casos propõe a transferência de um ramo do nervo radial para o nervo interósseo anterior (NIA), apresentando uma nova alternativa para o tratamento dessas lesões com resultados altamente satisfatórios.

**Objetivo:**

Demonstrar nossa estratégia, técnica e resultados na reinervação do NIA em lesões isoladas do tronco inferior do plexo braquial em quatro casos de lesão alta do nervo mediano.

**Método:**

Estudo de coorte prospectivo no qual quatro pacientes foram submetidos a neurotizações. O tratamento foi direcionado para a recuperação dos flexores dos dedos da mão e da preensão.

**Resultados:**

Todos os pacientes apresentaram reinervação do flexor pollicis longus (FPL) e dos flexores profundos do 2º, 3º e 4º dedo. O flexor profundo do 5º dedo também apresentou reinervação, porém com força reduzida (M3/4) em relação aos demais (M4+).

**Conclusão:**

Apesar do número de casos limitados neste e em outros estudos, os resultados se mostram uniformemente bons, o que permite considerar esse tratamento previsível. **Nível de Evidência IV, Série de Casos.**

## INTRODUCTION

Brachial plexus injuries mainly affect patients with polytrauma in high-energy accidents. In recent decades, with distal nerve transfers, including the procedure of transfer from the ulnar motor branch to the median nerve, popularly known as Oberlin,^1^ significant advances have been made in surgical options for the treatment of peripheral nerve lesions. However, most of the studies reported so far focused on upper trunk lesions.

The lower trunk lesion, known as Dejeri-klumpk, is uncommon, representing about 3-5% of brachial plexus lesions in adults.^2^ One of the functions lost in this specific group of patients is the flexion of the fingers, with important harming of the palmar grip, setting a great challenge for the surgeon. The literature has few reports for the treatment of lower trunk lesions. This study aims to demonstrate our strategy, technique, and results in the reinnervation of AIN in isolated lesions of the lower trunk of the brachial plexus in two cases and in two cases of high lesion of the median nerve.

### Objectives

Primary: to demonstrate our strategy, technique, and results in the reinnervation of the AIN in two cases of isolated lesions of the lower trunk of the brachial plexus and in two cases of high lesion of the median nerve.

Secondary: to demonstrate that the reinnervation of the AIN, contrary to common sense and most traditional textbooks, also promotes the reinnervation of the deep flexors of the 3rd, 4th, and 5th finger, in addition to the FPL and deep flexor of the 2nd finger, as described by Bertelli.^3^

## METHODS

This is a prospective cohort study in which four patients underwent neurotizations for AIN between April 2015 and May 2018. The treatment aimed to recover the flexors of the fingers of the affected hand, for recovery of the grip, and the treatment was clinically evaluated by the BRMC strength scale. Preoperatively, potential donor nerves were clinically tested, specifically the pronation and flexion of the wrist to the median nerve and the supination of the forearm and extension of the wrist, fingers, and thumb to the radial nerve.

### Description of surgical technique

An oblique “S” incision on the anterior aspect of the elbow between the brachioradial, proximal, and lateral muscle and the round, distal, and medial pronator muscle was performed. Fibrous lacertus was incised to identify the median nerve in the internal bicipital canal, along with the vascular bundle. The dissection of the median nerve extended from proximal to distal between the two heads of the round pronator muscle. Contrary to what Mackinon described, the pronator section was unnecessary for an adequate exposure of the median nerve and its branches. Only a release of the median nerve’s superficial fascia and forearm pronation was sufficient and less aggressive than the pronator section. The AIN was easily identified in all cases because it is the only lateral branch and has a path parallel to the median nerve.

**Figure 1:**
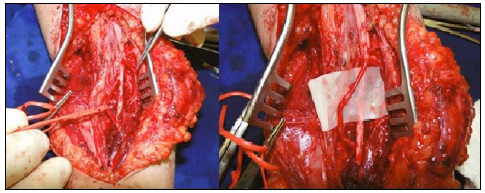
Identification and transfer of the branch of the round pronator to the anterior interosseous nerve.

Regarding low plexus injury, since part of the median nerve function was preserved (wrist flexion and forearm pronation), we planned the use of one of these branches as a donor nerve for the AIN. Proximal to the origin of the AIN, in the anteromedial portion of the median nerve, we identified the branch for the round pronator muscle and for the radial flexor of the carpal (Figure 1). Intraoperatively, the nerves were tested with a nerve stimulator (StimuplexHNS12; B. Braun Melsungen AG) with intensity between 0.5 and 2.0 mA. One of the pronator branches was distally sectioned and anastomosed to the AIN, which was proximally sectioned.

**Figure 2:**
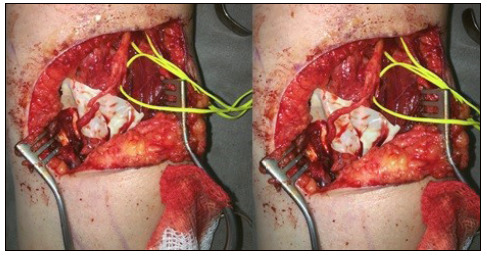
Identification and transfer of the branch of the short carpal radial extensor to the anterior interosseous nerve.

In cases of high lesion of the median nerve, we chose the motor branch of the radial nerve as donor for the Short Carpal Radial Extensor (SCRE). After locating the medial margin of the brachioradial, it was retracted to lateral, exposing the radial sensory nerve. This branch is then dissected proximally to its origin in the radial nerve. The motor branch of the radial nerve for the SCRE is identified between the sensory branch and the largest main terminal branch, which is the posterior interosseous nerve (Figure 2). These nerve branches were also stimulated with the same protocol mentioned above, to confirm if an adequate contraction was obtained and if the chosen branch was correct. The AIN was dissected as distally as possible, while the SCRE branch of the radial nerve was dissected and sectioned as proximally as possible, along with its origin in the radial nerve. The AIN stump was folded to proximal and lateral, while the branch of the SCRE was folded to distal. Neurorrhaphy was performed via an internal epineural suture without tension with nylon 10-0 with the aid of a microscope and reinforcement with fibrin glue (tissucol).

Postoperatively, the limb was immobilized with an axillopalmar splint, with elbow in flexion and mideum prono-supination for two weeks, followed by a short antebrachial splint for another two weeks to protect neurorrhaphy.

### Case 1

Male, nine years old, with a history of car trampling in August 2014, evolving to an injury on the lower trunk of the right brachial plexus. On the physical examination, seven months after the injury, he presented eyelid ptosis, normal shoulder and elbow function, active flexion and extension of the wrist, extension of the wrist and fingers M0, pronation M4/5, flexion of the thumb and fingers of the hand M0, intrinsic M0.

Surgery was performed nine months after the initial date of the injury. The initial plan was to transfer the pronator teres (PT) muscle branch to the AIN and supinator branch to posterior interosseous nerve (PIN). However, only the transfer of the PT branch to AIN was performed, because the PIN responded to the electrical stimulus in the intraoperative period.

At six months postoperative (PO), the patient presented a return of the M3 function of the FPL and Deep Flexor of the 2nd and 3rd finger and absence of active extension of the fingers. After 10 months of PO, this evolved to M4 strength of FPL and 2nd and 3rd finger Deep Flexor.

In the final follow-up, after three years of surgery, the patient presented M4+ strength of the FPL and Deep Flexor of the 2nd, 3rd, 4th, and 5th fingers, active extension with M4 strength of the extensors of the right hand fingers.

### Case 2

A 51-year-old patient with a history of schwannoma resection in the left axillary region, with a defect resulting from approximately 4 cm of the median nerve. Repair of the median nerve was performed with a graft of the lateral cutaneous nerve of the forearm at the same surgical time. After six months of PO, the patient maintained the flexion of the thumb fingers M0 and a neurotization of the branch of the SCRE muscle was performed for AIN.

Two months after the nerve transfer, the patient presented M3 force of the FPL and Deep Flexor of the 2nd, without any deficit in radial nerve function. After two years of follow-up, significant improvement in the strength of FPL and Deep Flexor of the 2nd, 3rd fingers M4+, and 4th and 5th fingers M3/4 were observed.

### Case 3

A 35-year-old patient with history of a lymph node biopsy in the left armpit. Intraoperatively, the surgeon found that it was a tumor of the lower trunk of the brachial plexus and opted for resection of the brachial plexus. In the anatomopathological postoperative, the diagnosis of schwannoma was confirmed. In the immediate postoperative period, the patient presented total paralysis of the superficial and deep flexors of all fingers and intrinsic muscles of the left hand, in addition to loss of strength (M2/3) of wrist flexors and pronators. The wrist and fingers extensors presented M3 strength and the triceps was normal. Shoulder and elbow function was not affected at any time. After 4 months of the initial injury, the strength of the extensors of the fingers and wrist, with Force M4/5, improved. However, the flexors of the fingers and the intrinsic of the hand did not recover. The strength of the wrist flexors and pronators remained in M3. At that moment, we performed the neurotization of the SCRE branch for AIN.

Three months postoperatively, the patient presented M3 strength of the FPL and flexors of the 2nd and 3rd finger, maintaining the extensor strength. In the last follow-up, after two years, the patient presented M4+ of the FPL and 2nd and 3rd fingers, and M4 of the 4th and 5th fingers.

### Case 4

A 66-year-old patient with a history of reverse prosthesis in the right shoulder presented a motor and sensory deficit in the territory of the median nerve. After four months of injury, the patient showed motor strength M0 of the FPL and the deep flexor of the 2nd finger, and M4 of 3rd to the 5th fingers, characteristic of a paralysis of the AIN. Wrist extenders and fingers remained the same. The patient showed no motor or sensory deficit in ulnar nerve territory.

After clinical follow-up for six months without improvement of the condition, the patient was subjected to the procedure of transfer branch of the SCRE to AIN (10 months after the injury). At two months after surgery, the patient presented motor strength M2/3 for FPL and deep flexor of the 2nd finger. After six months, the patient presented M4+ motor strength for FPL and Deep Flexor M4 of the 2nd finger.

## RESULTS

All patients presented FPL reinnervation, and deep flexors of the 2nd, 3rd, and 4th finger. The deep Flexor of the 5th finger also presented reinnervation, but with reduced force (M3/4) compared to the other fingers (M4+).

The mean time to observe the first visible contractions of the FPL and FDP of the 2nd, 3rd, 4th, and 5th finger was around three months, with progressive strength gain in the subsequent months (Table 1).

**Table 1:** Summary of cases, showing patients’ age, lesion etiology, time, and postoperative results.

Patient	Age	Etiology	Date of injury	Date of surgery	Pre op Physical examination	Pre op Physical examination	
**K/male**	15 years old	Collision bike vs. car	Aug 14	**April 2015** (Transfer branch of the round pronator to AIN).	**03/30/2015**	**02/06/2016**	**05/27/2019**
					Elbow 0 - 130	Elbow 0 - 130	Elbow 0 - 130
					Handle flex 80° ext 70°	Handle flex 80° ext 70°	Handle flex 80° ext 70°
					Flexion of fingers and thumb: absent	Flexion fingers and thumb: present GM4, extension of the fingers absent	Flexion fingers and thumb: present GM4, extension of the fingers absent
**S/female**	51 years old	Left armpit schwannoma	Nov 17	**November 2017** (schwannoma resection)	**05/10/2018**	**07/12/2018**	**01/30/2020**
					Elbow 0 - 130	Elbow 0 - 130	Elbow 0 - 130
				**May 2018** (Transfer of the SCRE branch to NIA)	Handle flex 80° ext 70°	Handle flex 80° ext 70°	Handle flex 80° ext 70°
					Flexion of thumb and finger: absent	Flexion of thumb and fingers: present GM4	Flexion of thumb and 2nd finger: present GM5
**I/female**	35 years old	Right armpit schwannoma	Jan 18	**May 2018** (Transfer of the SCRE branch to NIA)	**05/23/2018**	**08/30/2018**	**03/11/2020**
					Elbow 0 - 130	Elbow 0 - 130	Elbow 0 - 130
					Handle flex 80° ext 70°	Handle flex 80° ext 70°	Handle flex 80° ext 70°
					Flexion of thumb and finger: absent	Flexion of thumb and fingers: present GM3	Flexion of thumb and fingers: present GM3
**m/female**	66 years old	PO of shoulder arthroplasty	April 20	**Set 2020** (Transfer of the SCRE branch to AIN)	**09/03/2020**	**11/07/2020**	**03/25/2021**
					Elbow - 0 - 130	Elbow - 0 - 130	Elbow - 0 - 130
					Handle flex 80° ext 70°	Handle flex 80° ext 70°	Handle flex 80° ext 70°
					Handle flex 80° ext 70°		
					Flexion of thumb and finger: absent	Flexion of thumb and fingers: present GM2	Flexion of thumb and fingers: present GM5

We did not notice strength loss of donor nerves, round pronator, or wrist extensor in any case.

## DISCUSSION

Due to the poor functional prognosis of lower trunk lesions in adults, repair of the roots of C8 and T1 are not routinely indicated.^4^ In most cases, the emphasis of surgeons is on rebuilding the higher roots. Although isolated lesions of the lower trunk are much less frequent, they result in significant loss of motor function of the hand, since these lesions compromise the median and ulnar nerves.^3^ Similarly, in high lesions of the median nerve, post-traumatic or after tumor resection, although the nerve can be repaired with graft interposition, the results are poor in most cases, resulting in loss of grip.

The Anterior Interosseous Nerve (AIN) is the only branch that emerges on the lateral face of the median nerve, approximately 5 cm distal to the intercondylar line of the humerus. After the origin of AIN, there is a path parallel to the median nerve, located in the interval between the Flexor Pollicis Longus (FPL) and the Flexor Digitorum Profundus (FPD) innervating these two muscles. The AIN has a constant branch for the deep flexor of the indicator and partially innervates the deep flexor of the middle finger. However, Bertelli questions this knowledge and by direct clinical observation, he stated that the AIN also contributes to the innervation of the deep Flexor of the 3rd, 4th and 5th finger.^3^

Although most studies are based on small cases, distal nerve transfer focused on the reinnervation of the AIN, via motor branches of the radial (SCRE) or the preserved portion of the median nerve (PT, CRF), has shown promising results by several authors.^3^ ^),(^ ^5^ ^)-(^ ^8^

Mackninon and Novak^6^ report several advantages of the transfer of distal nerves to AIN, such as the proximity of motor plate receptors, elimination of the lesion area, and the guarantee of an axon source of a functioning donor nerve. Transferring a distal nerve close to the target muscle decreases the prolonged regeneration time, converting a proximal level lesion into a more distal one.^6^

We reported our results in a consecutive series of four patients treated with nerve transfer as a target to reinnervation of the AIN, aiming to restore the functions of the median nerve in the hand, especially the grip. The surgical procedure proved has low complexity and reproducible. All patients recovered flexion of all fingers, with significant improvement in grip function. Although the muscle strength obtained was higher in the FPL and deep flexor of the 2nd and 3rd fingers, the deep flexor of the 4th and 5th fingers exhibited functional recovery (M3/4) in all cases. The function of the deep flexor of the 5th finger was always inferior to the other fingers; however, it showed strength M3/4, thus, it was useful and satisfactory. Moreover, morbidity was minimal, since we observed no sequelae of donor nerves, either in the branches of the radial nerve or round pronator.

The time interval for recovery of FPL and deep flexor of the 2nd, 3rd, 4th, and 5th finger was surprisingly short, on average three months, with progressive gain of muscle strength during follow-up for up to two years after nerve transfer.

Other authors have also described similar experiences^3^ ^),(^ ^5^ ^)-(^ ^8^ with the reinnervation of the AIN. Bertelli’s study^3^ reports experiences in the reconstruction of thumb and finger flexion in four patients with extensive upper limb palsy due to high median nerve injury or C7-T1 brachial plexus avulsion, transferring the branch of the short carpal radial extensor (SREC) nerve to the anterior interosseous nerve (AIN) after eight months of surgery, all patients recovered total flexion of the fingers and thumb in an average period of 13 months postoperatively. The average grip strength was 5 kg and the tightening strength was 2 kg. Wrist extension was preserved in all patients. Despite residual sensory deficits, patients were able to use their hands regularly in daily life.

This technique should always be considered in lower brachial plexus lesions (C8-T1) or in upper lesions of the median nerve, for the restoration of thumb and finger flexion, when viable donor nerves are preserved, in particular the motor branch of PT or CRF or the branch of the SREC.

Restoration of thumb and finger grip function via nerve transfer of the SREC branch or PT to AIN proved to be a reliable and reproducible procedure. All patients recovered flexion of all fingers and not only FPL and FDP of the 2nd finger, with significant improvement in grip function.

## CONCLUSION

Despite the limited number of cases in this and other studies, the results were uniformly good, and this treatment may be predictable. Due to the relative rarity of these lesions, prospective multicentric studies should be encouraged to confirm the good reputation of this technique via results with better scientific evidence.
